# Whole genome characterization and evolutionary analysis of OP354-like P[8] *Rotavirus A* strains isolated from Ghanaian children with diarrhoea

**DOI:** 10.1371/journal.pone.0218348

**Published:** 2019-06-14

**Authors:** Susan Afua Damanka, Sabina Kwofie, Francis Ekow Dennis, Belinda Larteley Lartey, Chantal Ama Agbemabiese, Yen Hai Doan, Theophilus Korku Adiku, Kazuhiko Katayama, Christabel Chika Enweronu-Laryea, George Enyimah Armah

**Affiliations:** 1 Department of Electron Microscopy and Histopathology, Noguchi Memorial Institute for Medical Research, College of Health Sciences, University of Ghana, Legon, Ghana; 2 Department of Microbiology, School of Biomedical and Allied Health Science, College of Health Sciences, University of Ghana, Legon, Ghana; 3 Department of Virology II, National Institute of Infectious Diseases, Tokyo, Japan; 4 School of Basic and Biomedical Sciences, University of Health and Allied Sciences, Ho, Ghana; 5 Laboratory of Viral Infection I, Kitasato Institute for Life Sciences, Graduate School of Infection Control Sciences, Minato, Tokyo, Japan; 6 Department of Child Health, Korle Bu Teaching Hospital, College of Health Sciences, University of Ghana, Accra, Ghana; Stanford University School of Medicine, UNITED STATES

## Abstract

In 2010, the rare OP354-like P[8]b rotavirus subtype was detected in children less than 2 years old in Ghana. In this follow-up study, to provide insight into the evolutionary history of the genome of Ghanaian P[8]b strains RVA/Human-wt/GHA/GHDC949/2010/G9P[8] and RVA/Human-wt/GHA/GHM0094/2010/G9P[8] detected in an infant and a 7-month old child hospitalised for acute gastroenteritis, we sequenced the complete genome using both Sanger sequencing and Illumina MiSeq technology followed by phylogenetic analysis of the near-full length sequences. Both strains possessed the Wa-like/genotype 1 constellation G9P[8]b-I1-R1-C1-M1-A1-N1-T1-E1-H1. Sequence comparison and phylogenetic inference showed that both strains were identical at the lineage level throughout the 11 genome segments. Their VP7 sequences belonged to the major sub-lineage of the G9-lineage III whereas their VP4 sequences belonged to P[8]b cluster I. The VP7 and VP4 genes of the study strains were closely related to a Senegalese G9P[8]b strain detected in 2009. In the remaining nine genome segments, both strains consistently clustered together with Wa-like RVA strains possessing either P[8]a or P[8]b mostly of African RVA origin. The introduction of a P[8]b subtype VP4 gene into the stable Wa-like strain backbone may result in strains that might propagate easily in the human population, with a potential to become an important public health concern, especially because it is not certain if the monovalent rotavirus vaccine (Rotarix) used in Ghana will be efficacious against such strains. Our analysis of the full genomes of GHM0094 and GHDC949 adds to knowledge of the genetic make-up and evolutionary dynamics of P[8]b rotavirus strains.

## Introduction

Diarrhoea remains one of the leading causes of death in children younger than five years. In 2015, nearly 500, 000 children died as a result of diarrhoea [1]. Rotavirus A (RVA), a member of the genus *Rotavirus* and family *Reoviridae* is a leading cause of viral diarrhoea in children under five years. Despite the availability of safe and effective rotavirus vaccines such as Rotarix by GlaxoSmithKline Biologicals and Rotateq by Merck & Co. Inc., in 2016, rotavirus was responsible for an estimated 128, 500 deaths of children under the age of five, of which 104, 733 deaths occurred in sub-Saharan Africa [2].

The rotavirus particle is a triple-layered, non-enveloped icosahedron enclosing an 11-segment genome of double-stranded RNA (dsRNA) which codes for six structural (VP1 to VP4, VP6 and VP7) and six non-structural proteins (NSP1 to NSP5/6) [3]. Rotaviruses are commonly classified based on the genes expressing the two outer capsid proteins VP7 (glycoprotein; G) and VP4 (protease sensitive protein; P) [3]. At least 36 G genotypes and 51 P genotypes have been reported in humans and animals so far (https://rega.kuleuven.be/cev/viralmetagenomics/virus-classification/rcwg). Of the numerous G and P genotypes that have been reported, six G and P genotype combinations are commonly detected among human RVA strains; they are G1P[8], G2P[4], G3P[8], G4P[8], G9P[8], and G12P[8] [4, 5].

Within the last decade, the genome classification of RVA strains has been extended to include the remaining nine internal capsid and non-structural protein genes. In this regard, the complete genome of RVAs i.e. VP7-VP4-VP6-VP1-VP2-VP3-NSP1-NSP2-NSP3-NSP4-NSP5/6 is denoted by the descriptor Gx-P[x]-Ix-Rx-Cx-Mx-Ax-Nx-Tx-Ex-Hx where x indicates genotype number, respectively [6–8]. There are currently 36G, 51P, 26I, 22R, 20C, 20M, 31A, 22N, 22T, 27E, and 22H genotypes approved by the Rotavirus Classification Working Group (https://rega.kuleuven.be/cev/viralmetagenomics/virus-classification), however, most human RVA strains are classified into three genotype constellations namely the Wa-like/genotype 1 (G1/G3/G4/G9-P[8]-I1-R1-C1-M1-A1-N1-T1-E1-H1), DS-1-like/genotype 2 (G2 -P[4]-I2-R2-C2-M2-A2-N2-T2-E2-H2) and AU-1-like/genotype 3 (G3-P[9]-I3-R3-C3-M3-A3-N3-T3-E3-H3) [6, 9]. Of these three constellations, the Wa-like strains are the most predominant followed by the DS-1-like strain while the AU-like strains are the least detected in humans globally.

Currently, the P[8] genotype of the VP4 gene is classified into two genetically distinct subtypes, P[8]a and P[8]b (OP354-like) [10, 11]. Most of the P[8] strains belong to subtype P[8]a, which are commonly detected globally, whilst P[8]b strains though detected globally, are rare. The P[8]b RVA strains have gained attention recently since they seem to be spreading globally after they were first reported in Africa [10–15]. Even though they were first reported in Malawi [10], their origin has been traced by phylogeographic studies to Asia with a subsequent spread to other continents [15]. Unfortunately, the actual global prevalence of P[8]b is largely unknown because the commonly used P-genotyping primers fail to detect them. Therefore at least the VP8* nucleotide sequence of the VP4 gene is required to determine the P[8] variant.

It is common knowledge that P[8] strains normally possess the Wa-like constellation and therefore plausible to expect P[8]b strains to naturally carry the Wa-like genotype constellation. However, it is noteworthy that P[8] bearing strains such as G1P[8]and G3P[8] possessing the DS-1-like backbone genes emerged and spread globally in recent years [16–32].

Most previous studies describing P[8]b strains [10–15] have characterised only the outer capsid genes (VP7 and VP4), excluding the ‘backbone’ genes. In our previous study, we reported the detection of five P[8]b RVA strains in children who were hospitalized due to rotavirus gastroenteritis in 2010 [12]. In the present study, we successfully characterised the complete genome of two of the Ghanaian P[8]b RVA strains by Sanger sequencing method and Illumina MiSeq sequencing technology to determine the complete genotype constellation and to provide insight into the evolutionary dynamics of their genes by employing maximum likelihood phylogeny and the Bayesian evolutionary analysis by sampling trees (BEAST).

## Materials and methods

### Ethical approval

The study was approved by the Institutional Review Board, Noguchi Memorial Institute for Medical Research, University of Ghana, Legon, Ghana.

### Study strains

The strains in this study RVA/Human-wt/GHA/GHM0094/2010/G9P[8] and RVA/Human-wt/GHA/GHDC949/2010/G9P[8] were detected by Enzyme Linked Immunosorbent Assay and polyacrylamide gel electrophoresis in samples obtained from an infant and a 7-month old child, respectively. They were partially characterised (VP7/VP4 genes) by reverse transcription polymerase chain reaction and Sanger sequencing [12].

### RNA extraction and full genome sequencing by Sanger method

Total viral RNA was extracted from 500 μL of 10% stool suspension by the phenol/chloroform method described by Steele and Alexander [33]. The RNA was further purified with an RNaid kit (MP Biomedicals, LLCQ BioGene) following manufacturer’s instructions. Prior to reverse transcription of the viral genome, 8 uL of RNA was denatured at 94°C for 5 minutes and quickly chilled on ice. Complementary DNA (cDNA) was synthesized at 42°C for 60 minutes using Pd(N) hexamer nucleotide random primers and the avian myeloblastosis virus reverse transcriptase kit (Promega corporation, Madison, WI, USA) following the manufacturer’s recommendations.

The amplification strategy for the 11 genes of the RVA strains was based on previously published primers [34], some of which were modified (S1 Table) based on results of *in silico* PCR performed using a global collection of sequences from Wa-like and DS-1-like strains as template.

The 11 gene segments were each amplified from the cDNA using the GoTaq G2 DNA polymerase kit (Promega corporation, Madison, WI, USA) using gene-specific primers (S1 Table) [initial denaturation at 94°C, 2 min; followed by 40 cycles of PCR at 94°C, 1 min, 51°C, 1min, 72°C, 3min; final extension at 72°C, 5 min]. The successfully amplified genes were purified using EXOSAP-IT (USB products Affymetrix, Cleveland, OH USA) following manufacturer’s protocol. The purified amplicons were sequenced in the forward and reverse directions using the BigDye Terminator v3.1 cycle sequencing kit (Applied Biosystems, USA). Post-sequencing purification was performed using the ethanol/sodium acetate precipitation method and sequences subsequently read using an ABI 3130xl automated genetic sequence analyser (Applied Biosystems, USA). The forward and reverse sequences generated were assembled and genotypes were assigned using the online genotyping tool for RVA strains RotaC v2.0 [35] and the Virus Pathogen Resource (ViPR) [36].

### Full genome sequencing using the Illumina MiSeq technology

Aliquots of the extracted RNA were sent to the National Institute of Infectious Diseases, Tokyo, Japan, for full genome characterization. cDNA library preparation and Illumina MiSeq sequencing were performed as previously described [31, 37], with modifications. Briefly, a 200 bp fragment library ligated with bar-coded adapters was constructed for each sample with the NEBNext Ultra RNA Library Prep Kit for Illumina v1.2 (New England Biolabs, Ipswich, MA), according to the manufacturer’s instructions. cDNA libraries were purified with Agencourt AMPure XP magnetic beads (Beckman Coulter, Brea, CA), and their quality assessed on a MultiNA MCE-202 bioanalyzer (Shimadzu Corporation, Kyoto, Japan). Nucleotide sequencing was performed on an Illumina MiSeq sequencer (Illumina, San Francisco, CA) using the Illumina MiSeq Reagent Kit v2 (300 cycles) to generate 151 paired-end reads.

### Sequence and phylogenetic analysis

#### Sequence assembly

Sequence assembly was carried out using CLC Genomics Workbench v7.0.3 (CLC Bio). Contigs were assembled from the obtained sequence reads by *de novo* assembly. The genotype of each viral gene segment was determined using ViPR [36].

#### Phylogenetic analysis: Maximum likelihood phylogenetic analysis

Using the Basic Local Alignment Search Tool (BLAST) and the rotavirus resource in virus variation available at the NCBI website [38, 39], reference sequences for phylogenetic analysis were retrieved. Multiple sequence alignment was carried out on the datasets followed by the determination of the best fit nucleotide sequence substitution models using MEGA version 6.06 [40]. Using the best fit nucleotide substitution models with the lowest Bayesian Information Criterion scores [41], i.e. HKY+G (VP1), TN93+I (VP2, NSP3), GTR+G+I (VP3), TN92+G (VP4, VP6, VP7, NSP1, NSP2, NSP4, NSP5), maximum likelihood phylogenetic trees for all the eleven genome segments were constructed with 1000 bootstrap replicate trials.

#### Phylogenetic analysis: Determination of the time to the most recent common ancestor of the Ghanaian P[8]b strains using Bayesian evolutionary analysis by sampling trees (BEAST)

To provide insight into the time when the G9P[8]b strains analysed in this study were introduced into the Ghanaian population, the BEAST method was employed and the time to the most recent common ancestor (tMRCA) of the P[8]b VP4 sequences and G9 VP7 sequences were computed. The Bayesian Markov chain Monte Carlo (MCMC) method implemented in BEAST v1.8.2 [42] was used. An uncorrelated lognormal relaxed-clock model [43], a coalescent constant size tree prior [44] together with the best-fit nucleotide substitution models (based on Bayesian Information Criterion) were used. The MCMC calculations were carried out for 4.5 x 10^7^ generations for the VP4 sequences and 10 x 10^7^ for the VP7 sequences. The BEAGLE library was used to enhance the computational speed of the analysis [45]. The results as examined by the Tracer software v1.6 showed effective sampling sizes of ≥200. The maximum clade credibility trees were annotated with the TreeAnnotator (v1.8.1) and viewed in FigTree.

#### Nucleotide sequence accession numbers

Near-complete genome nucleotide sequences obtained for strains GHM0094 and GHDC949 were deposited in to the DDBJ/GenBank/EMBL database under the accession numbers LC439261- LC439282 (Table 1).

**Table 1:** Nucleotide sequence accession numbers, lengths of genes obtained from Illumina MiSeq sequencing and closest strain based on phylogeny.

	RVA/Human-wt/GHA/GHDC949/2010/G9P[8]b		RVA/Human-wt/GHA/GHM0094/2010/G9P[8]b				
Gene	Accession number	Length of gene sequenced/bp (%)	Accession number	Length of gene sequenced/bp (%)	Nt identity between GHDC949 & GHM0094 (%)	Closest strain in the phylogenetic tree (% nt identity)	
VP1	LC439261	3293 (99.7)	LC439262	3291 (99.7)	99.9	RVA/Human-wt/SEN/MRCDPRU2051/2009/G9P[8]	(99.9)
VP2	LC439263	2727 (100.0)	LC439264	2724 (100.0)	100	RVA/Human-wt/MLI/Mali-133/2008/G1P[8] RVA/Human-wt/MLI/Mali-033/2008/G1P[8]	(99.5) (99.5)
VP3	LC439265	2582 (99.7)	LC439266	2582 (99.7)	99.8	RVA/Human-wt/MLI/Mali-136/2008/G1P[6]	(99.7)
VP4	LC439267	2352 (99.7)	LC439268	2321 (98.4)	99.8	RVA/Human-wt/SEN/MRCDPRU2051/2009/G9P[8]	(99.3)
VP6	LC439269	1343 (99.0)	LC439270	1346 (99.3)	99.9	RVA/Human-wt/MLI/Mali-021/2008/G1P[8]	(99.8)
VP7	LC439271	1041 (98.0)	LC439272	944 (88.9)	100	RVA/Human-wt/SEN/MRCDPRU2051/2009/G9P[8]	(100)
NSP1	LC439273	1554 (99.2)	LC439274	1554 (99.2)	99.8	RVA/Human-wt/SEN/MRCDPRU2051/2009/G9P[8]	(99.8)
NSP2	LC439275	1015 (95.9)	LC439276	1046 (98.8)	99.8	RVA/Human-wt/GHA/Ghan-005/2008/G1P[8]	(99.7)
NSP3	LC439277	1072 (99.8)	LC439278	1070 (99.6)	100	RVA/Human-wt/SEN/MRC-DPRU2051/2009/G9P[8]	(99.9)
NSP4	LC439279	741 (98.8)	LC439280	721 (96.1)	99.8	RVA/Human-wt/ZAF/1723WC/2009/G9P[8]	(99.4)
NSP5	LC439281	651 (98.0)	LC439282	647 (97.4)	99.9	RVA/Human-wt/SEN/MRC-DPRU2051/2009/G9P[8]	(99.8)

## Results

### Genotype constellation of the study strains

The near full length open reading frames of the 11 genome segments of strains GHM0094 and GHDC949 were successfully determined using the Illumina MiSeq technology. The lengths obtained ranged from 88.9–100% and are shown in Table 1. The genotype constellation of both strains was G9-P[8]b-I1-R1-C1-M1-A1-N1-T1-E1-H1—a typical Wa-like genetic backbone possessed by Wa-like strains reported elsewhere in the world (Table 2).

**Table 2:** Genotype constellation of reference Wa-like and study strains.

	Genotype constellation											
Strain	VP7	VP4	VP6	VP1	VP2	VP3	NSP1	NSP2	NSP3	NSP4	NSP5	References
RVA/Human-wt/USA/Wa/1974/G1P[8]	G1	P[8]a	I1	R1	C1	M1	A1	N1	T1	E1	H1	[6]
RVA/Human-tc/USA/P/1974/G3P[8]	G3	P[8]a	I1	R1	C1	M1	A1	N1	T1	E1	H1	[6]
RVA/Human-wt/USA/IAL28/1992/G5P[8]	G5	P[8]a	I1	R1	C1	M1	A1	N1	T1	E1	H1	[6]
RVA/Human-wt/USA/WI61/1983/G9P[8]	G9	P[8]a	I1	R1	C1	M1	A1	N1	T1	E1	H1	[6]
RVA/Human-wt/GHA/GHDC949/2010/G9P[8]	G9	P[8]b	I1	R1	C1	M1	A1	N1	T1	E1	H1	This study
RVA/Human-wt/GHA/GHM0094/2010/G9P[8]	G9	P[8]b	I1	R1	C1	M1	A1	N1	T1	E1	H1	This study

### Whole genome sequence comparison

**Fig 1:**
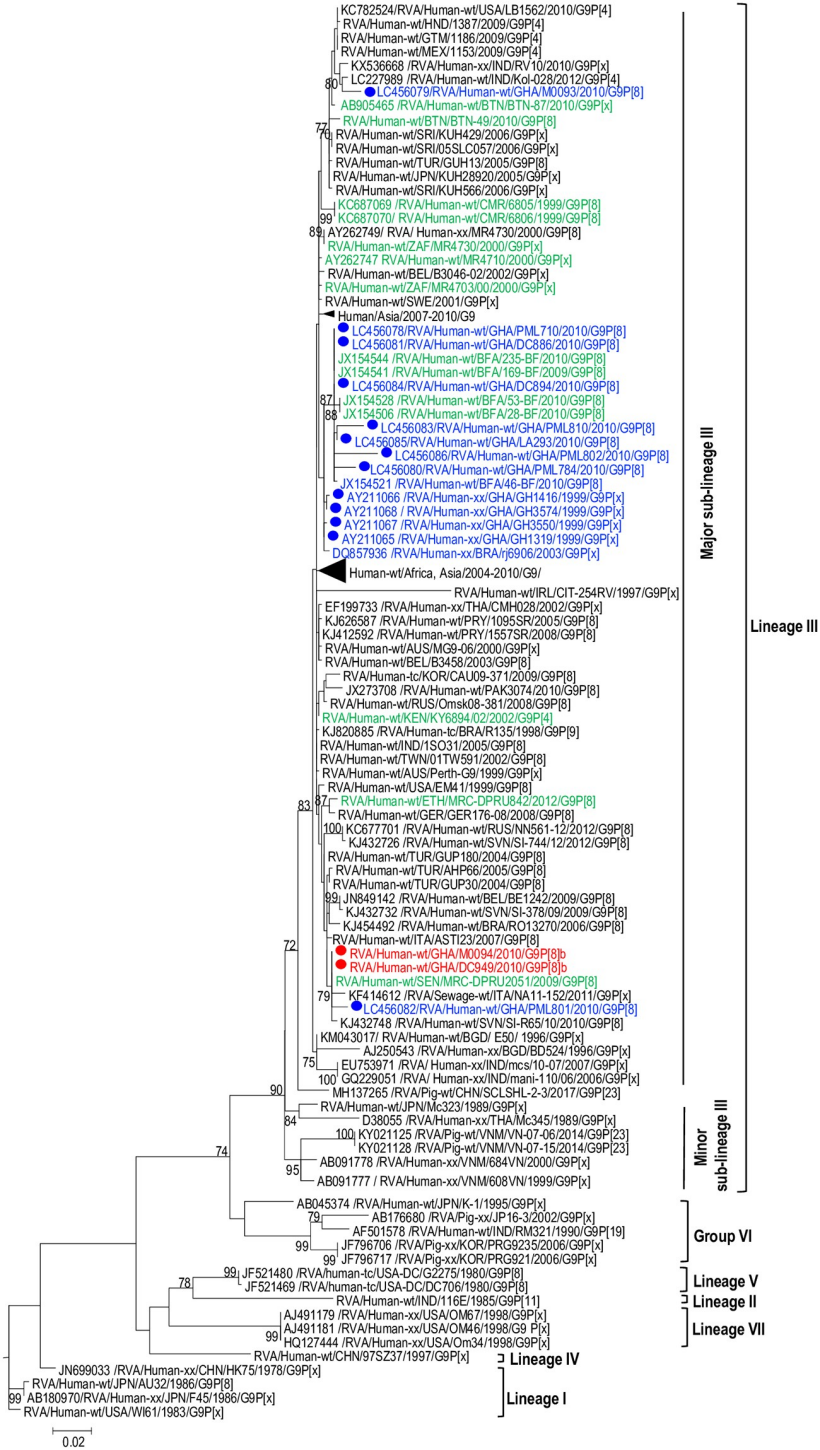
Phylogenetic trees of the outer capsid protein gene (VP7) of G9 strains. The study strains (indicated in red dots) were analysed alongside published RVA strains. Maximum likelihood phylogenetic analysis was performed using the best-fit nucleotide substitution TN92+G. Bootstrap values (1000 replicates) of ≥ 70% are shown. Scale bar indicates genetic distance expressed as number of nucleotide substitutions per site.

**Fig 2:**
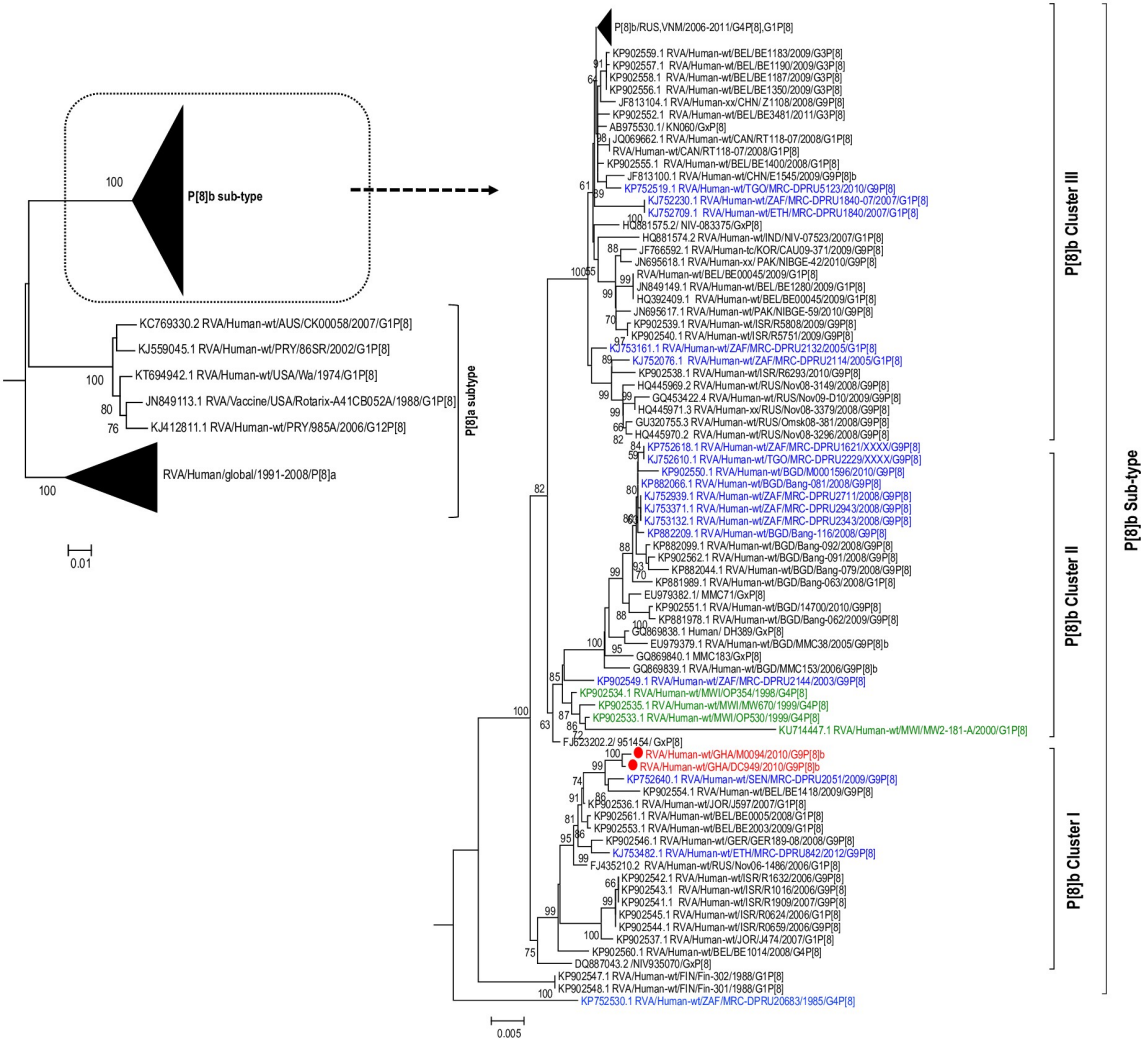
Phylogenetic trees of P[8] VP4 sequences. The study strains (indicated in red dots) were analysed alongside published RVA strains. Maximum likelihood phylogenetic analysis was performed using the best-fit nucleotide substitution TN92+G. Bootstrap values (1000 replicates) of ≥ 70% are shown. Scale bar indicates genetic distance expressed as number of nucleotide substitutions per site. African strains are in blue font whereas the OP354-like strains detected in Malawi are indicated in green font.

**Fig 3:**
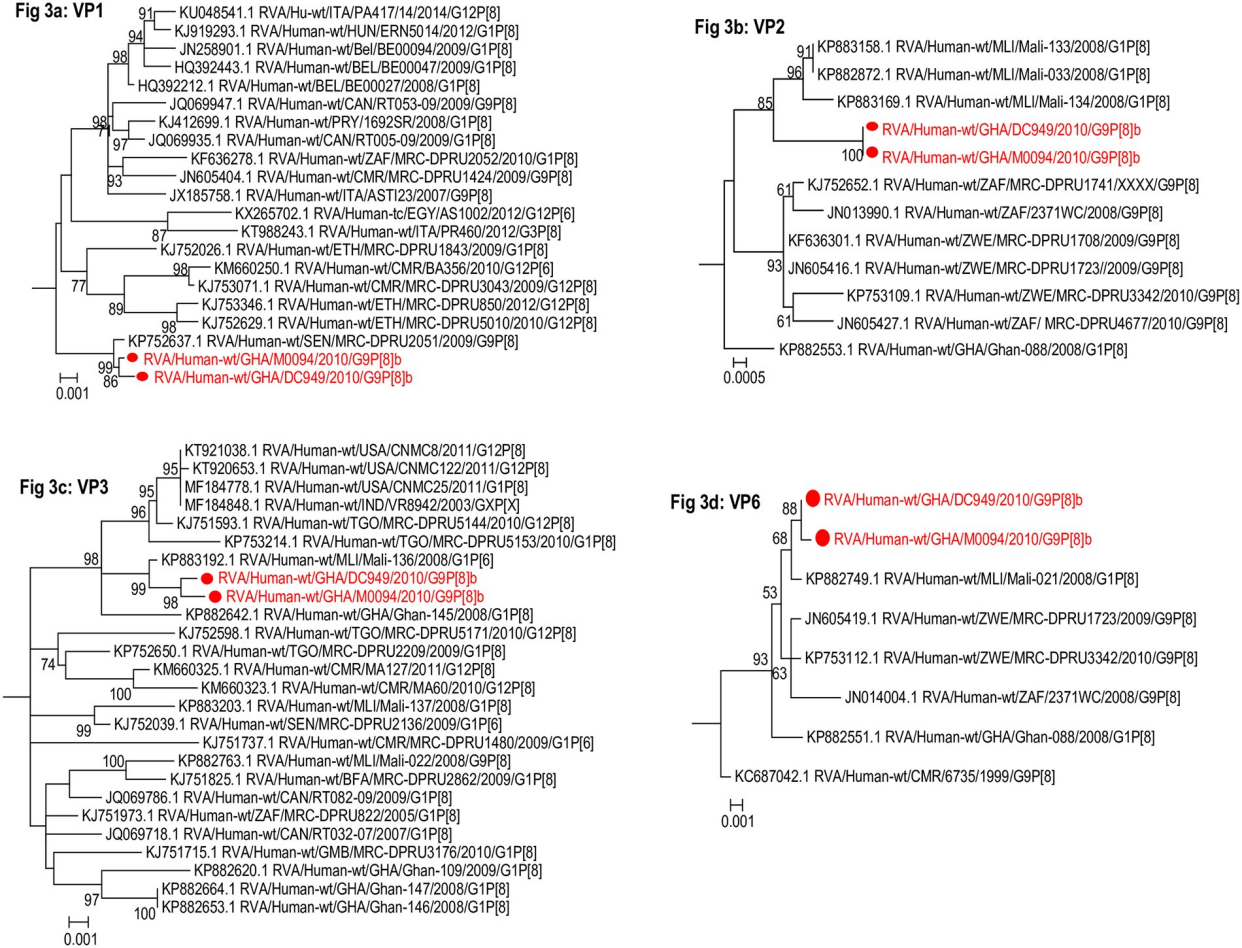
Phylogenetic trees of the internal and middle layer protein genes of Wa-like (genotype 1) strains: a) VP1 b) VP2 c) VP3 and d) VP6. Trees shown are sub-trees. The study strains (indicated in red dots) were analysed along with published human RVA strains. Maximum likelihood phylogenetic analysis was performed using the best-fit nucleotide substitution models. Bootstrap value (1000 replicates) of ≥ 70% are shown. Scale bar indicates genetic distance expressed as number of nucleotide substitutions per site.

**Fig 4:**
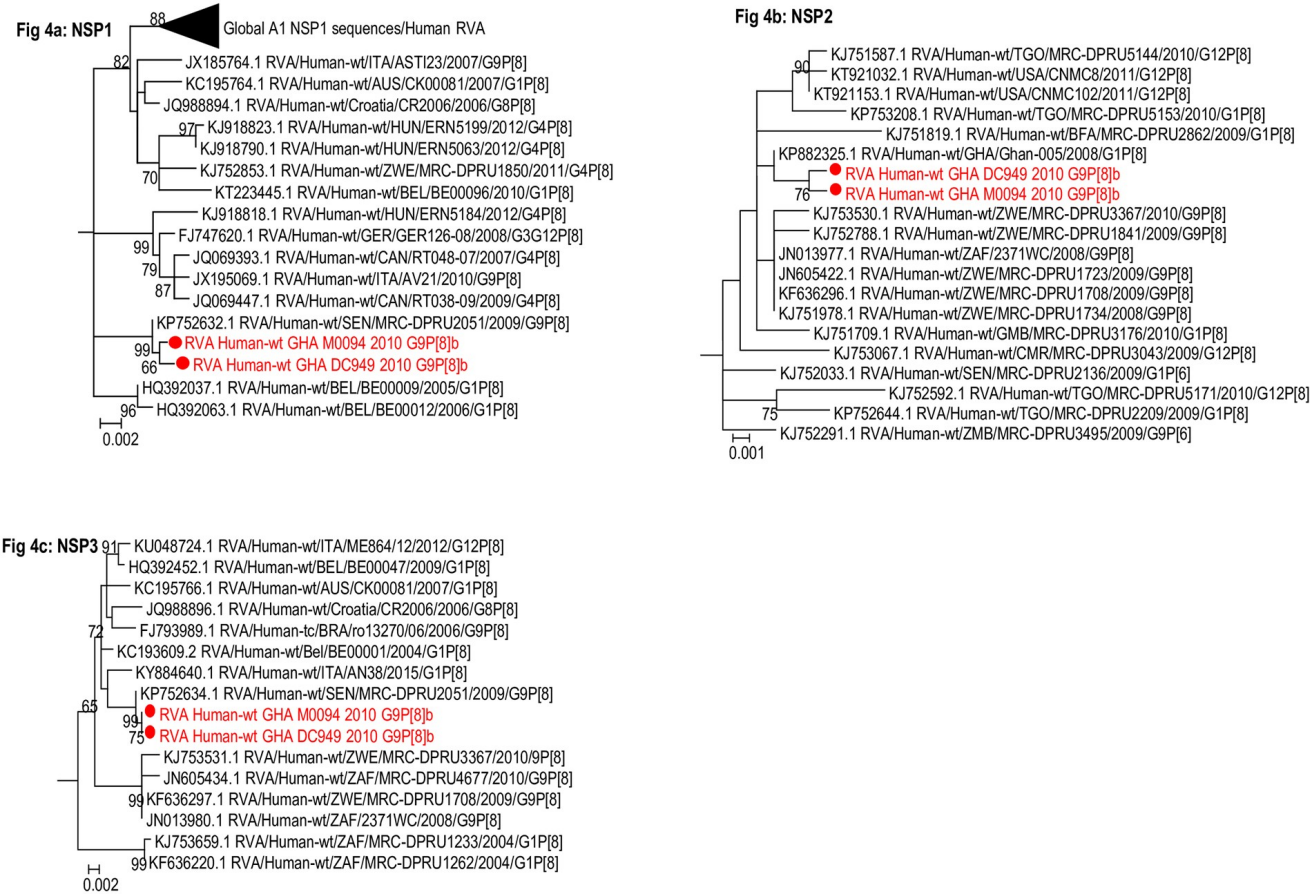
Phylogenetic trees of the non-structural protein genes of Wa-like (genotype 1) strains. a) NSP1 b) NSP2 c) NSP3. Trees shown are sub-trees. The study strains (indicated in red dots) were analysed along with published human RVA strains. Maximum likelihood phylogenetic analysis was performed using the best-fit nucleotide substitution. Bootstrap value (1000 replicates) of ≥ 70% are shown. Scale bar indicates genetic distance expressed as number of nucleotide substitutions per site.

**Fig 5:**
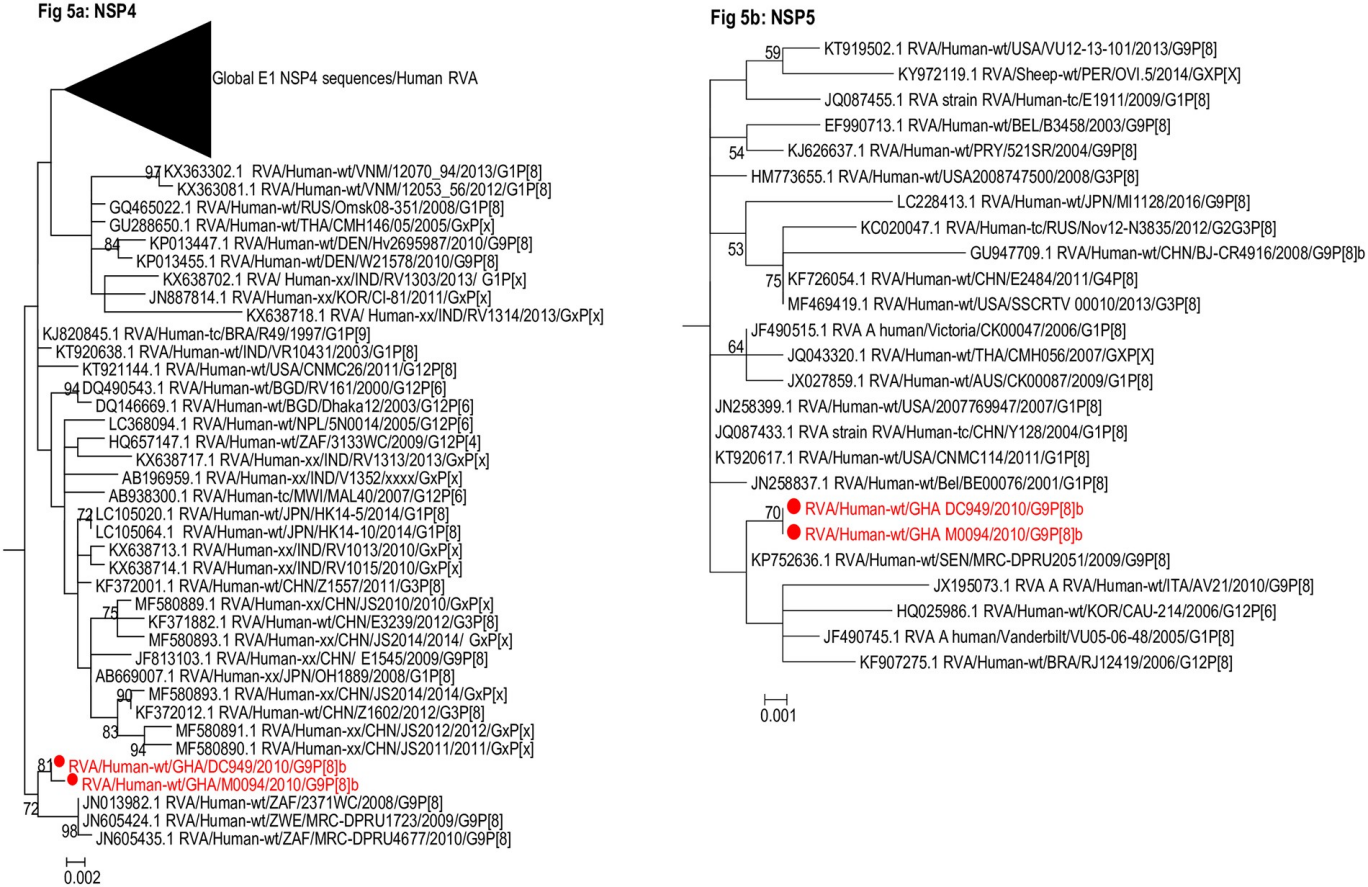
Phylogenetic trees of the non-structural protein genes of Wa-like (genotype 1) strains. a) NSP4 b) NSP5. Trees shown are sub-trees. The study strains (indicated in red dots) were analysed along with published human RVA strains. Maximum likelihood phylogenetic analysis was performed using the best-fit nucleotide substitution. Bootstrap value (1000 replicates) of ≥ 70% are shown. Scale bar indicates genetic distance expressed as number of nucleotide substitutions per site.

The two Ghanaian G9P[8]b strains characterised in this study were identical across the 11 genome segments Table 2. Their nucleotide sequence identities for the 11 genes ranged from 99.8% to 100% (Table 1). The two study strains were also consistently highly identical to contemporary typical Wa-like strains detected during 2008–2009 from the African continent (Table 1; Figs 1–5). Specifically, six genes—structural protein genes VP7, VP4, VP1, and the non-structural proteins genes NSP1, NSP3 and NSP5 of the study strains were closest to a Senegalese G9P[8] strain MRC-DPRU2051 detected in 2009. Although the five remaining genes namely VP2, VP3, VP6, NSP2, and NSP4 of the study strains did not apparently share the same closest neighbour, they too were closely related to contemporary Wa-like G1P[8] or G9P[8] RVA strains detected on the African continent around 2008 and 2009 (Figs 1–5, Table 1). The average nucleotide sequence identity between the study strains and their closest strains ranged from 99.4–99.8% (Table 1).

### Phylogenetic inference of GHDC949 and GHM0094

#### The outer capsid genes: VP4 and VP7

The VP7 gene of the study strains belonged to the major sub-lineage of the G9-lineage III (Fig 1)—a lineage to which majority of the contemporary G9 RVA strains of human host species origin belong. Phylogenetic analysis of a global collection of P[8]b strains classified them into three distinct large clusters designated Cluster I, Cluster II, and Cluster III (Fig 2). This observation was consistent with Zeller et al.’s report on global P[8]b sequences in 2015 [15].

Cluster I contained mainly G1P[8] and G9P[8] strains from Israel, Belgium, Jordan, Ethiopia, Senegal, and Ghana (this study), and a G2P[8] strain from India detected in the early 1990s. Cluster II contained the prototype OP354-like G4P[8] strains/1999 from Malawi (indicated in green font) as well as G9P[8] strains from Bangladesh, South Africa and Togo (Fig 2). Cluster III contained G1P[8], G3P[8], G4P[8], G9P[8] strains from Asia, America, Europe, and Africa detected from 2005–2012. In addition to these three major clusters, two old Finish strains RVA/Human-wt/FIN/Fin-302/1988/G1P[8] and RVA/Human-wt/FIN/Fin-301/1988/G1P[8] forming a monophyletic cluster, shared a common ancestor with the ancestral sequence from which the above mentioned major clusters diverged. Noteworthy is a South African G4P[8] strain RVA/Human-wt/ZAF/MRC-DPRU20683/1985/G4P[8] which could qualify as the progenitor of all P[8]b strains (considering the data available in GenBank database) based on the topology of the P[8]b tree. This strain diverged first from the ancestral root of the P[8]b strains followed by the Finish strains and then cluster I, cluster II, and cluster II. In the present study, the Ghanaian P[8]b sequences (GHDC949 and GHM0094) belonged to Cluster I where they formed a monophyletic cluster with African and European G9P[8] strains RVA/Human-wt/SEN/MRC-DPRU2051/2009/G9P[8] and RVA/Human-wt/BEL/BE1418/2009/G9P[8] with a 99% bootstrap support (Fig 2). The only other P[8]b strain from Africa within cluster I was detected in Ethiopia in 2012. Our study strains were clearly distinct from the prototype OP354-like strains detected in Malawi in 1999 which rather belonged to cluster II in the phylogenetic tree (indicated in green font).

**Fig 6:**
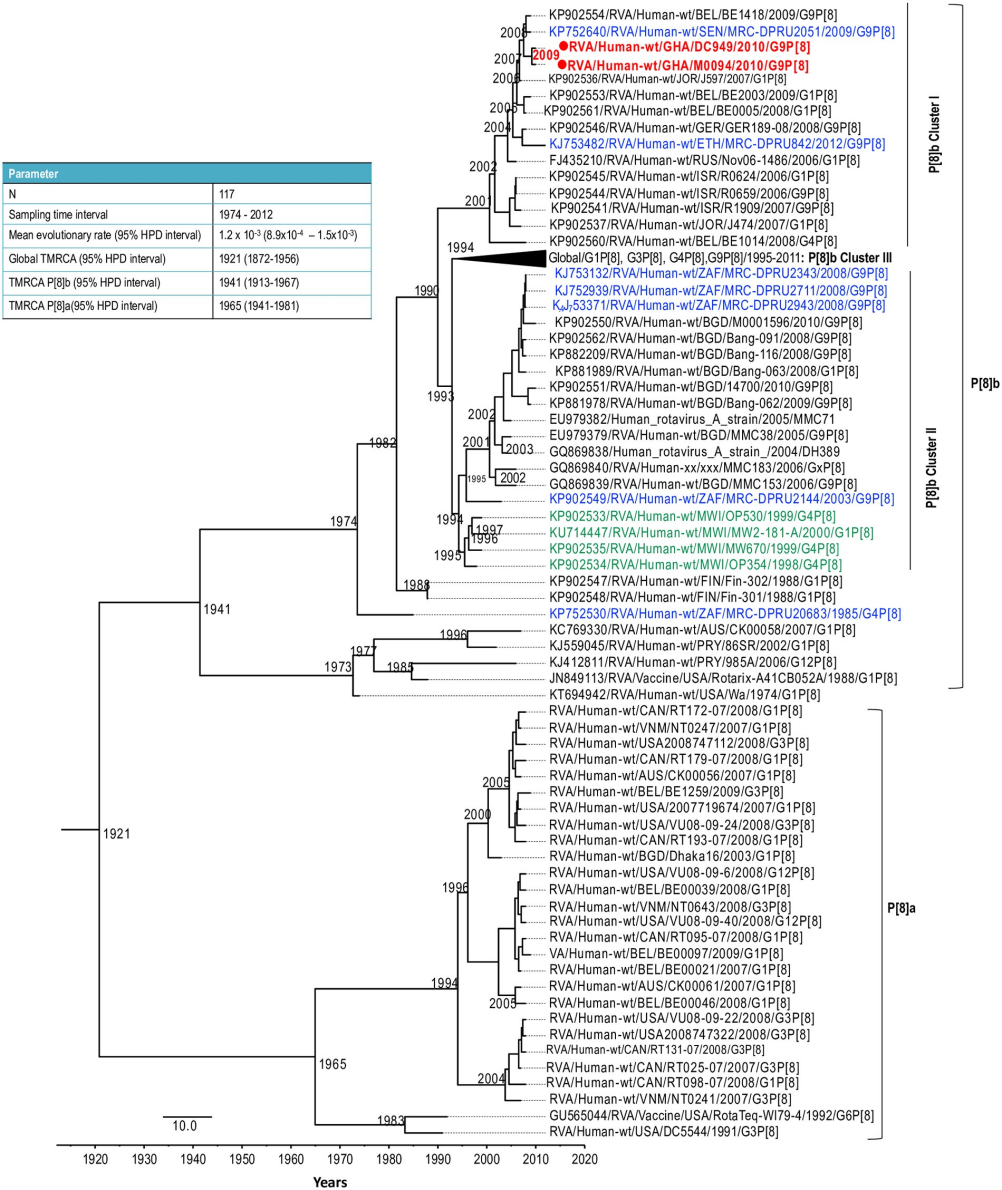
A simplified maximum clade credibility trees for the P[8] VP4 in this study together with a global collection of P[8]a and P[8]b sequences of RVA strains detected from 1974–2012. Strains from this study are indicated in red fonts and red dots. Other African strains are in blue font whereas the OP354-like strains detected in Malawi are indicated in green font.

**Fig 7:**
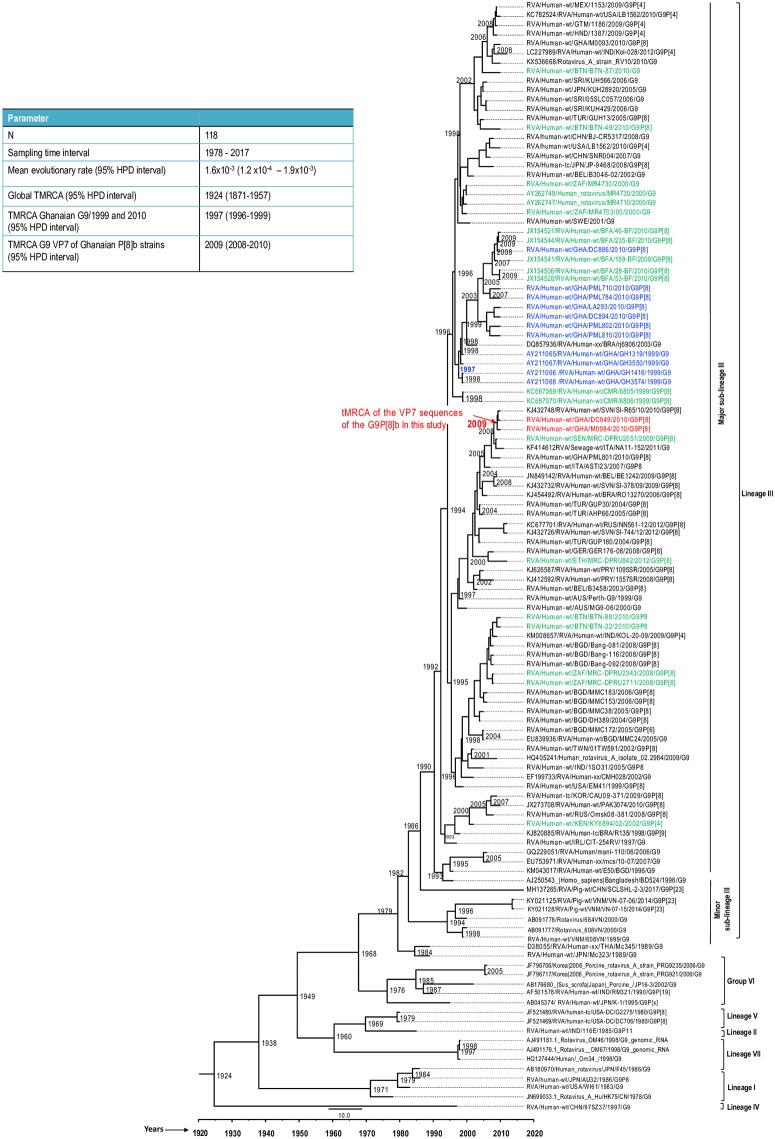
A simplified maximum clade credibility trees for the G9 VP7 sequences of strains characterized in this study together with a global collection of G9 strains of both human and porcine RVA strains detected from 1978–2017. Strains from this study are indicated in red fonts and red dots. Other African strains are in green font whereas previously published Ghanaian G9 strains are indicated in blue font.

BEAST analysis revealed that the P[8]b sequences diverged from a common ancestor shared with P[8]a sequences around the year 1921 (Highest posterior density (HPD) interval: 1872–1956) (Fig 6). The most recent common ancestor of the two Ghanaian P[8]b sequences reported in this study existed around the year 2009 (Highest posterior density (HPD) interval: 2008–2010). In addition, the most recent common ancestor of the G9 VP7 sequences of the two strains together with a Ghanaian G9 strain RVA/Human-wt/GHA/PML801/2010/G9P[8]a and a Senegalese strain RVA/Human-wt/SEN/MRC-DPRU2051/G9P[8] was estimated to be around 2007 (Highest posterior density (HPD) interval: 2008–2010) (Fig 7). It is worth noting that the tMRCA of the cluster that contained some historical and contemporary Ghanaian G9 sequences reported in 1999 [46] and 2010 (GenBank data; accession numbers: LC456078- LC456081, LC456083- LC456086; strains indicated in blue font) was 1997 (HPD interval: 1997–1999) and this cluster was distinct from the cluster containing the two study strains.

#### Internal capsid and non-structural protein genes: VP1-3, VP6, NSP1-5

The high nucleotide sequence identity of the internal capsid and non-structural protein genes of our study strains to African Wa-like strains was supported by the clustering pattern of the sequences upon phylogenetic analysis. The two study strains consistently clustered together with sequences of RVA strains from Africa mostly forming a monophyletic cluster supported by high bootstrap values (Figs 3–5).

## Discussion and conclusion

The rare P[8]b VP4 gene subtype has long been reported to be distinct from the P[8]a subtype; however, there is limited information on the relationships between the backbone genes of the P[8]b and P[8]a RVA strains. In this study, with the complete genome sequences of two previously published P[8]b strains from Ghana—GHM0094 and GHDC949, we showed by phylogenetic analyses that their backbone genes were typical Wa-like RVA genes which are carried by the globally common P[8]a RVA strains. Our observation was consistent with the single previous report on full genomic analyses of P[8]b strains [47] as well as Kuzuya et al. [13] who reported that the VP6, VP7, and NSP4 genes of Japanese G1P[8]b strains were highly homologous to G1P[8]a strains prevalent in the same area.

Of note was the consistently high similarity observed between many genes of a Senegalese G9P[8]b strain MRC-DPRU2051 and our study strains. In this regard, we propose that perhaps, the P[8]b strains analyzed in this study were generated by reassortment event(s), where regionally rare P[8]b strains such as the Senegalese MRC-DPRU2051-like strains, reassorted with G9-Wa-like RVA strains circulating in Ghana, donating their VP4 and other genes. The time of the most recent common ancestor estimated for the VP4 gene and the VP7 gene of our study strains by BEAST analysis showed that the ancestral VP7 and VP4 sequences of these two genes existed around 2007 and 2009. This evidence together with the year of detection of the Senegalese G9P[8]b strain, lend a support to the above proposition.

It has been suggested that introducing a single or small number of genetically distinct gene segment(s) into well-adapted human rotaviruses can result in a progeny virus with a genetic composition that is well-suited for replication and spread in the human population [48]. Such introductions of a P[8]b subtype VP4 gene into the stable Wa-like strain backbone may result in strains that might propagate easily in the human population, with a potential to become an important public health concern, especially because it is not certain if the monovalent rotavirus vaccine (Rotarix) used in Ghana will be efficacious against such strains.

In summary, the genomes of GHM0094 and GHDC949 consisted of the P[8]b subtype in combination with a contemporary Wa-like genetic backbone not different from the backbone genes of the globally encountered P[8]a strains. Also, the 11 genes of the study strains shared common ancestors with RVA genes of African origin. Our analysis of the full genomes of GHM0094 and GHDC949 add to knowledge of the genetic make-up and evolutionary dynamics of P[8]b rotavirus strains. To be able to detect the gradual spread of such P[8]b genes in the commonly detected regional strains, it is important to continuously monitor the genome sequences of not only the unusual RVA strains but also the common RVA strains as they might be acquiring variants of the commonly detected genotypes which can only be deciphered at the nucleotide and lineage level.

## Supporting information

S1 Table OP354-like primers for PCR and sequencing. (DOCX)
