# Supplementary material for: Whole genome characterization and evolutionary analysis of OP354-like P[8] Rotavirus A strains isolated from Ghanaian children with diarrhoea
Source: PLoS One. 2019 Jun 14;14(6):e0218348. doi: 10.1371/journal.pone.0218348 (PMC6570025; doi:10.1371/journal.pone.0218348)
Supplement: S1 Table — (DOCX) [file pone.0218348.s001.docx]

**Table S1: OP354-like primers for PCR and sequencing**

| **Gene** | **Primer Name** | **Sequence (5' - 3')** |
| --- | --- | --- |
| VP1 | VP1 C-0011f | GCTGTACAATGGGGAAGTA |
|  | VP1 C(0)R | GGTCACATCTAAGCRCTCTAA |
|  | VP1(R1-2)-1468f | GCRAARCAYACYAGAGARTA |
|  | VP1(R1-2)-1487r | TAYTCTCTRGTATGYTTTGC |
|  | VP1 C-2147r | GCTTGATCCCATTGTGTACTTTG |
|  | VP1(R1)-2804r | GATGATTTTGACCCACTATCTTCTAT |
| VP2 | VP2-5prime | GGCTATTAAAGGCTCAATGG |
|  | VP2 C (-29)R | TACAGTTCGTTCATRATGCG |
|  | VP2(C1)-644f_m | GATGCTGGKAAAGTTGTAGATTC |
|  | VP2(C1-2)-2225r | ATCCRTACATCTCATCTCTTTC |
| VP3 | VP3 C-0019f | AGTAGTGYGTTTTACCTCTG |
|  | VP3 C (0)R | GGTCACATCRTGACTAGTGTGTTA |
|  | VP3(M1-2)-1336r | TACACCTTTTGGTATRAATCTTTTAA |
|  | VP3C-1995r | TCATAATATYTTCCACCTTC |
| VP4 | VP4 C-011f | TGGCTTCGCTCATTTATAGACA |
|  | VP4 C (+3)Ad-R | GGGGGTCACATCCTC |
|  | VP4(P4_8)-1653r_m | CATTACRCTAGTYGCCATTGA |
|  | VP4(P4_8)-2028r | TCGATAYGATCGTTTTGGAATAA |
| VP6 | VP6 C (0)F | GGCTTTAAAACGAAGTCTTC |
|  | VP6 C (0)R | GGTCACATCCTCTCACT |
| VP7 | VP7 C (0)F | GGCTTTAAAAGMGAGAATTTCC |
|  | VP7 C (+3)Ad-R | GGGGGTCACATCATACAATTCT |
| NSP1 | NSP1 F | GGCTTTTTTTATGAAAAGTCTTGTG |
|  | NSP1 C (-18)rev | CTAGGCGCTACTCTAGT |
| NSP2 | NSP2-5prime | GGCTTTTAAAGCGTCTCAGTC |
|  | NSP2-3prime | GGTCACATAAGCGCTTTCTATTC |
| NSP3 | NSP3-5prime | GGCTTTTAATGCTTTTCAGTGGTTG |
|  | NSP3-3prime | GGTCACATAACGCCCCTATAG |
| NSP4 | NSP4 C-003f | CTTTTAAAAGTTCTGTTCCGAGAG |
|  | NSP4 C-743r | TAAGACCATTCCTTCCATTAAC |
| NSP5 | N5-5pri | GGCTTTTAAAGCGCTACAGT |
|  | N5-3pri | GGTCACAAAACGGGAGTGGGGA |
